# Sensemaking by Employees in Essential versus Non-essential Professions During the COVID-19 Crisis: A Comparison of Effects of Change Communication and Disruption Cues on Mental Health, Through Interpretations of Identity Threats and Work Meaningfulness

**DOI:** 10.1177/08933189221087633

**Published:** 2022-05

**Authors:** Ward van Zoonen, Ronald E. Rice, Claartje L. ter Hoeven

**Affiliations:** 1 Erasmus School of Social and Behavioral Sciences (ESSB), 6984 Erasmus University Rotterdam, Rotterdam, The Netherlands; 2 Department of Communication, University of California Santa Barbara, Santa Barbara, CA, USA

**Keywords:** sensemaking, change communication quality, essential and non-essential work, identity threat, mental health, meaningful work

## Abstract

This study examines the implications of categorizing workers into essential and non-essential groups due to disruptions in work associated with—and the quality of organizational change communication about—the COVID-19 pandemic. Specifically, we examine how these cues trigger identity threats and influence the meaningfulness of work, consequently affecting the mental health of workers (anxiety, distress, and depression). The results show that change communication reduces identity threat, while also increasing meaningfulness of work, for both work categories. However, the disruptions increase identity threat only for non-essential workers. Conversely, identity threat increases two of the three mental health issues while meaningfulness of work reduces two of them. The study contributes to our growing understanding of the pervasive, though subtle, implications of COVID-19 for the workplace by showing how a process of employee sensemaking and organizational change communication directly and indirectly influence important dimensions of mental health.

Pandemics (e.g., black plague in 1350, Spanish Flu 1918, SARS 2003) and extreme events (e.g., war, terrorism, natural disasters) have historically shaped work and organizing (Morgeson et al., 2015). The COVID-19 pandemic and associated outbreak response strategies—including social distancing, remote work mandates, and the categorization of work as essential or non-essential—have sparked speculation about the impact of the crisis on the role of work in employees’ lives, and on employee wellbeing (Kramer & Kramer, 2020; Spurk & Straub, 2020). This study seeks to understand how employees engage in sensemaking regarding cues of work categorization, work disruption, and organizational communication about COVID-19 related changes, and how that sensemaking affects their mental health.

Organizational sensemaking involves individuals first seeking out and interpreting (environmental) cues, then developing plausible interpretations of equivocal and uncertain (often unexpected) events and issues, and finally changing or reinforcing procedures (Maitlis, 2005; Maitlis & Christianson, 2014; Weick et al., 2005), such that workers can continue to manage their expectations, work, and responsibilities. In this study these cues are the categorization of workers into essential and non-essential workers due to COVID policies, the disruptions from COVID they experience in their work, and the quality of change communication about COVID provided by the organization. We seek to explore the ways in which these cues trigger identity threats and affect perceptions of meaningful work, and ultimately, how these interpretations affect employees’ mental health.

## Organizational Sensemaking and COVID-19

Organizational sensemaking is a pervasive human activity that aids in ascribing meaning to events in employees’ surroundings (Angeli & Montefusco, 2020; Crayne & Medeiros, 2020; Stephens et al., 2020). Organizational sensemaking theory aims to understand how organizations operate as interpretive systems (Maitlis & Christianson, 2014) and to elucidate how the framing of decisions in event sequences guides inferences and behaviors (Cornelissen et al., 2014). Sensemaking studies have frequently explored how violated expectations (e.g., threats to organizational identity; Dutton & Dukerich, 1991) represent cues that trigger sensemaking (Maitlis & Christianson, 2014). The experienced discrepancy and its significance are contextual and subjective, and as such sensemaking and resulting interpretations may be triggered to varying extents across work contexts. Wrzesniewski et al.’s (2003) account of interpersonal sensemaking explains how the meaning of work is constructed through direct or subtle behavioral and interpersonal cues in the workplace. Interpretation of these cues may affect perceptions of the meaning of work and ultimately responses (e.g., mental health) to those altered meanings.

Sensemaking under pressure, including natural disasters and global health crises such as COVID-19, especially requires employees (and organizations) to make timely and swift decisions, as these events may have ambiguous and uncertain outcomes, the stakes are high, and the decisions consequential (Cornelissen et al., 2014; Morgeson et al., 2015; Quinn & Worline, 2008; Weick, 1995). These events may disrupt employees’ workflow, challenging their understanding of the world and creating uncertainty about how to act (Maitlis & Christianson, 2014). Sensemaking in such a context has been studied in relation to organizational crises (e.g., Bhopal gas leak; Weick, 2010), terrorism (e.g., hijacking of United Airlines Flight 93; Quinn & Worline, 2008), environmental shocks (Milliken, 1990), and the COVID-19 outbreak (Stephens et al., 2020).

Sensemaking is an ongoing, interactive process in which individuals seek or experience cues, assign meaning, and move to action with one another (Thomas et al., 1993), influencing the perception and enactment of professional identities (Cheney et al., 2014). Organizations, partially through their managerial communication, can shape meaning making through sensegiving; i.e., providing relevant interpretations and goals of a change to affected employees (Gioia & Chittipeddi, 1991). van den Heuvel et al. (2009) define meaning-making as “the ability to integrate challenging or ambiguous situations into a framework of personal meaning using conscious, value-based reflection” (p. 508).

Notably, this study is not concerned with internal processes of enactment and organizing (as emphasized by Weick, 1995). Instead, we focus on a process in which individuals perceive and then interpret environmental cues, leading to consequential outcomes (here, wellbeing) (Maitlis & Christianson, 2014). As Weick et al. (2005, p. 417) note, the sensemaking literature may exaggerate agency (“construct” and “enact”) while underemphasizing responding (“react” and “comply with”). Hence, in the COVID-19 context, we consider work categories, disruptions in work, and change communication quality as cues that are interpreted in terms of identity threat and meaningfulness of work, which ultimately affect employees’ mental health, as part of a general sensemaking process.

## Review and Hypotheses

### Cues for Sensemaking during COVID-19

#### Work Categories

Studies have highlighted the importance of occupational status during the pandemic, albeit related to employment status (Spurk & Straub, 2020). Previously, during the SARS pandemic of 2003, research concluded that essential workers—that is, workers in system-relevant occupations—reported particularly high levels of emotional distress (Maunder et al., 2006). Stephens et al. (2020) speculate that categorizations of essential versus non-essential work may influence sensemaking processes about organizational identification, as well as job satisfaction. Thus, we suggest that work categorizations provide different cues for the sensemaking process. In the Netherlands, the context for our study, the formal distinction between essential and non-essential work did not exist before COVID-19, but was an important part of coordinating outbreak response strategies. This distinction helped determine not only who should stay at home and who should continue their work as before, but also, for instance, whether or not their children could still go to day care facilities. The publication of such a categorization and its central role in the outbreak response created a new context where occupational status mattered more.

#### Disruptions in Work

Organizations and employees try to make sense of disruptions of work routines associated with a major crisis. These disruptions include changes in employment, procedures, schedules, and coordination and organization of work. Though it is hard to imagine any work that remain unaffected by the COVID-19 pandemic, different jobs and occupational groups are likely not affected similarly. For instance, workers may vary in how they experience the COVID-19 pandemic, as some may be positively affected (less contact with bullying colleagues, greater ability to focus on work), while others may experience negative implications (more isolation, identity threats) (Spurk & Straub, 2020).

#### Change Communication Quality

Various scholars have examined the interdependencies of communication and change in the context of organizational sensemaking (Ford & Ford, 1995). Organizations may inform employees in various ways, and to various extents, about associated organizational changes. Change communication is an important occasion for sensemaking, as sensemaking involves the attribution of meaning to a target (e.g., events) through the placement (framing) of this target into a mental model (Cornelissen et al., 2014; Weick, 1995). Activating a frame through organizational change communication may create expectations about important aspects of the context by directing a specific or default elaboration. Such communication provides cues about how to make sense of the change. Unplanned change, certainly exemplified by the COVID-19 pandemic, creates uncertainty and confusion, generating a sensemaking process, and possibly leading to stress, negative feedback, and disengagement (Li et al., 2021). Internal, transparent organizational communication—involving relevant, needed, substantial, accurate, timely, and balanced information, along with employee participation and organizational accountability during crises—can substantially affect how employees interpret, manage, and cope with such changes (Li et al., 2021).

Internal crisis communication management involves the use of multiple channels to provide necessary information, opportunities for discussion, emotional resources, and acknowledgements of the employees’ contributions and concerns (Heide & Simonsson, 2019). A survey of nearly 1,000 employees in different organizations in Germany during COVID-19 emphasized the importance of frequent communication, provision of detailed and substantive content, participation in the communication, and openness (Ecklebe & Löffler, 2021). Such high-quality communication improved relationships between employees and their organizations. Another study of over 1,000 employees in multiple organizations in Austria showed that internal informational communication (measured as relevant, timely, complete, understandable, accurate, and reliable communication) during COVID-19 was positively associated with reception of managerial decisions (Einwiller et al., 2021). Analyzing responses from nearly 500 full-time U.S. employees during April 2020, Li et al. (2021) showed how transparent internal communication reduced employees’ change-related uncertainty, aided their ability to cope with the associated stress, and improved organization-employee relationships. Internal organizational crisis communication in the form of sense-giving and meaning-making discourse by leaders in two U.K. universities helped employees make sense of the implications of the COVID-19 health crisis (Yeomans & Bowman, 2021). Such communication involved core narratives of organizational resilience and competence; empathy, reassurance, and recognition; and aspects of location and community. The core narratives reduced uncertainty and fostered belongingness and organizational identification.

### Interpretations of Cues and Relationships to Mental Health

In this research, we consider two manifestations of sensemaking interpretations of the cues: threats to identity and meaningfulness of work.

#### Identity Threats

Strong organizational identities may help employees deal with stressful situations and perceive those situations as less threatening. *Identity threats* are situations in which central, distinctive, and enduring organizational characteristics are challenged, triggering strong reactions from organizational members (Ran & Golden, 2011). Disruptions to routines may threaten the security and coherence of an identity (Thatcher & Zhu, 2006). When work itself is threatened, the reactions may be particularly negative (Berjot et al., 2013). Research suggests that identity threat may increase mental health issues (Rothausen et al., 2017). Given the centrality of work in constructing one’s identity (Berjot et al., 2013), it is reasonable to assume that threats to one’s work identity may also present challenges to overall wellbeing (Lazarus & Folkman, 1984).

Research on employee sensemaking during organizational change has suggested that sensemaking may be anchored by frames relying on identity, culture, and structure. In addition, research on sensemaking and blame during disasters indicates that discourses of identity emerged while employees engaged in sensemaking (Gephart, 1993). The seminal study by Dutton and Dukerich (1991) of the Port authority discussed how sensemaking of events could threaten organizational identities by highlighting the discrepancy between an organization’s identity and its image. Similarly, Ravasi and Schultz (2006) analyzed how managers in competitive environments were prompted to make sense of organizational identities by answering questions such as “is this who we really are?” In the context of remote work, one study concluded that organizational change was “first and foremost a challenge to their [employees’] identity; hence their sensemaking involved crafting a new sense of self” (Bean & Eisenberg, 2006, p. 216), and that employees who articulated claims on fixed identity labels (e.g., who I am) experienced distress in transitioning to nomadic work.

*Change communication* may help to reduce identity threats. When identities are called into question, providing a consistent narrative might help organizational members attach meaning to events, issues, and actions (Ravasi & Schultz, 2006). As such, organizations (i.e., their leaders) should reconstruct and communicate a consistent narrative and emphasize positive elements to help organizational members rebuild their sense of who they are as part of the organization. As another example, when telecommuting becomes mandatory (for instance to cut costs or address security issues), the change may have a negative impact on organizational identities (Kurland & Bailey, 1999), mainly ascribed to a lack of efficient identity-related communication (Thatcher & Zhu, 2006). Corley and Gioia (2004) demonstrated that confusing messages from the organization and restrictions on communication prevented employees from making sense of new organizational identities. Research has reported the importance of (vertical) communication in strengthening the distinctiveness of organizations in order to aid identification (Agarwal & Buzzanell, 2015; Scott, 2007). Identity threat may also occur when employees feel devaluated, unappreciated, or insignificant. Thus, it is important for the organization and managers to adequately communicate about the disruption to reassure that the disruption is not a reflection of the employees’ worth or value, and not an identity threat. Thus:

H1a1: Disruption of work is positively associated with identity threat among workers.
H1a2: This relationship is stronger for those newly categorized as non-essential workers than for essential workers.
H2a: Change communication quality is negatively associated with identity threat.
H3: Identity threat is positively associated with mental health issues—that is, H3a anxiety, H3b distress, and H3c depression.

### Meaningfulness of Work

Individuals yearn for meaning in their lives; increasingly work has become a source of such meaning (Martela & Pessi, 2018; Steger et al., 2012). In its broadest sense, meaningfulness refers to the significance and value of work (Lips-Wiersma et al., 2016), although others see meaningful work as being more about pursuing a purpose (beyond money) (Sparks & Schenk, 2001) or as a sense of return on investment in terms of physical, cognitive, and emotional energy (Kahn, 1990; for other conceptualizations, see Allan et al., 2019; Bailey et al., 2019). Meaningful work has also been associated with values such as doing work that is morally worthy (Ciulla, 2011). In this study, we view meaningful work as a subjective experience, or evaluation, of one’s work (Martela & Pessi, 2018). Meaningful work, therefore, is not what work means to people (meaning), but employees’ evaluation of the significance and positive valence of their work (Steger et al., 2012), where positive valence has a eudaimonic (growth- and purpose oriented) focus. A lack of meaningful experiences can be a serious psychological deprivation associated with reduced well-being (Martela & Pessi, 2018).

There are two dominant conceptualizations of meaningful work; unidimensional and multidimensional conceptualizations. Although multidimensional operationalizations are sometimes criticized for capturing sources of variance beyond the meaningfulness construct (Allan et al., 2019), unidimensional measures of meaningfulness often fail to capture the complexity of meaningfulness factors (Lips-Wiersma & Wright, 2012). In one multidimensional approach, Steger et al. (2012) proposed three dimensions central to experiencing work: psychological meaningfulness (PM), meaning making through work (MM), and greater good motivations (GG). Briefly, PM reflects the subjective experience of workers by capturing the sense that people judge their work to matter and be meaningful. MM is linked to the ways in which meaningful work experiences can benefit people’s overall meaning in life. Finally, GG reflects the idea that work is most meaningful when it is has a broader impact on others, beyond the self.

Employees strive to actively construct meaning (Carton, 2018; van den Heuvel et al., 2009), in particular about ongoing work experiences, by interpreting relevant sensemaking cues (Aguinis & Glavas, 2019). Through sensemaking, employees can search for and find meaningfulness in organizational change efforts, because sensemaking allows them to engage in work behaviors that matter, are significant to others inside and/or outside the organization, and can serve a greater good (PM, MM, and GG) (Aguinis & Glavas, 2019). Meaningfulness of work amid organizational change can be viewed as the employee-construed sense of specific organizational change significance, importance, and worth (Shulga, 2021).

*Change communication* is important in the process of constructing meaning and meaning convergence (Shulga, 2021). Efforts to promote change through open and effective communication is believed to increase meaning making (Sonenshein & Dholakia, 2012; van den Heuvel et al., 2013) and perceptions of meaningfulness of work (Leiter & Harvie, 1998). Even in work that may be considered rote, communication of the organization’s position and aspirations can help to increase meaningfulness (Carton, 2018).

Sensemaking aids the extent to which employees are effective in maintaining a sense of purpose and meaningfulness (van den Heuvel et al., 2013), especially during times of organizational and environmental change. Building on conservation of resources theory (Hobfoll, 2001), research suggests that sensemaking through observing and interpreting information that they receive from their environment can be a resource used by employees to find meaning in, and engage with, change (van den Heuvel et al., 2009; van den Heuvel et al., 2013). Although a lack of meaningfulness of one’s work can lead to disengagement and alienation (Aktouf, 1992), a sense of meaningfulness in one’s work is likely to improve adaptability to change (Weick, 1995) and thus mental health (Allan et al., 2019).

However, the meaningfulness of work could be considerably affected when organizations, and society at large, change the status of some occupations, such as explicitly valuing or labeling some work and occupations as more essential than others (Steger et al., 2012). For instance, Kramer and Kramer (2020, p. 2) noted that “changes in the status of different occupations can alter individuals’ perceptions regarding the three dimensions [of] meaningful work.” Hence, we argue that some occupational groups (here, essential work vs. non-essential) may be differentially affected by the pandemic in terms of meaningfulness of work. Therefore, we hypothesize:

H1b1: Disruption of work increases perceptions of meaningful work.
H1b2: This relationship will be stronger for those newly categorized as essential workers than for non-essential workers.
H2b: Change communication quality is positively related to perceptions of meaningful work.
H4: Perceptions of work as meaningful is negatively associated with mental health issues—that is, H4a anxiety, H4b distress, and H4c depression.

### Indirect Effects

The above hypotheses imply several (conditional) indirect effects. The first set concerns indirect effects of work disruption. Thus:

H5a1: Disruption of work is positively related to mental health issues, through identity threat.
H5a2: This relationship will be stronger for those newly categorized as non-essential workers than essential workers.
H5b1: Disruption of work is negatively related to mental health issues, through perceptions of work as meaningful.
H5b2: This relationship will be stronger for those newly categorized as essential workers than non-essential workers.

The second set concerns indirect effects of change communication. Thus:

H6a: Change communication quality is negatively related to mental health issues, through identity threat.
H6b: Change communication quality is negatively related to mental health issues, through perceptions of work as meaningful.

**Figure 1.**
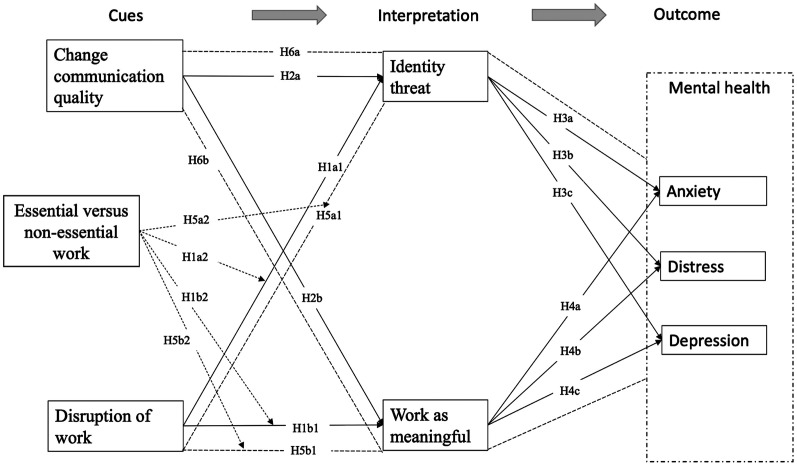
Hypothesized relationships. *Note:* Dashed lines with arrowhead represent moderation hypotheses, dashed lines represent indirect relationships, solid lines indicate direct effects.

Figure 1 portrays the concepts, relationships, and hypotheses.

## Method

### Sample and Procedures

Data were collected in the first weeks of the COVID lock-down measures and the non-essential/essential work distinctions announced by the Dutch government. The study analyzes survey data provided by 623 Dutch employees: 321 employees were recruited from non-essential sectors and 302 employees from essential sectors (defined below). Dynata, a panel research firm, recruited the respondents who were qualified based on screening questions including (non)essential work, employment status, work hours, and sectors. Respondents failing attention or duration checks were not accepted. Although approximately 45% of the Dutch workforce works in essential sectors,^1^ we aimed to sample an equal number of respondents from essential and non-essential sectors. The sample is otherwise representative of Dutch working adults in terms of age, gender, and education.

The respondents were on average 42.3 years old (SD = 12.95), and 49.8% was female and 50.2% male. The average work week consisted of 34 hours (SD = 9.97) and the average tenure was 10.4 years (SD = 10.43). Most workers in essential sectors were employed in health care (48.5%), education (18.3%), and food supply (16.9%). Non-essential workers worked in trade and private services (15.8%), public services (14.6%), non-essential health care (12.4%), and science and research (11.2%). We also asked respondents to indicate their level of job insecurity on a seven-point scale; non-essential workers (*M* = 2.67, *SD* = 1.16) reported slightly but significantly higher levels of insecurity than essential workers (*M* = 2.30, *SD* = 1.20; Δ*M* = 0.372, *t* = 3.929, *df* = 614, *p* < .001). However, it should be noted that respondents overall did not seem too worried about potential job loss, with an average concern of 2.49 (SD = 1.19) on a seven-point scale.

### Measures

*Change communication quality* was measured using six items adapted from Bordia and colleagues (2004). Participants were asked to evaluate the communication their employer has provided about their organization’s response to COVID-19 in terms of usefulness, adequacy, positive communication, appropriateness, timeliness, and accuracy, measured from 1=strongly disagree to 7=strongly agree.

*Disruption of work* was measured using six items adapted from Akgün et al. (2006). Originally, the items represented unlearning behavior, referring to changes in work routines. These measures were modified to reflect individual changes in work routines during the COVID-19 crisis. Respondents were prompted to consider the extent to which specific work activities were affected by the ongoing COVID-19 pandemic: “to what extent...” “have information sharing mechanisms changed?,” “has your ability to make decision changed?,” “has the timing of your work changed?,” “has the way you coordinate your work changed?,” “have your work hours changed?,” and “has the physical location of your work changed?,” measured from 1=not at all to 7=changed completely.

*Essential* versus *non-essential work*. Respondents were screened based on the recent categorization of work into essential and non-essential types, as part of the Dutch government’s policies, as of March 16, 2020, as a response to COVID-19.^2^ We double checked this classification by asking respondents to indicate whether they worked in essential or non-essential professions. This led to respondents being labelled as non-essential (=0) or essential (=1) sector employees.

*Identity threat* was measured using five items adapted from Berjot et al. (2013) representing the *Threat to Social Group Identity* (TSGI) subscale to assess perceived threat to the positivity and distinctiveness of social identity (Tajfel & Turner, 1985). In the context of this study, we prompted respondents to think about implications of the current health pandemic for their occupational group. Items include “I had the feeling that the members of my occupational group including myself were totally not appreciated,” measured from 1=strongly disagree to 7=strongly agree.

*Perception of meaningfulness of work* was assessed by adopting 10 items from the work as meaning inventory (WAMI; Steger et al., 2012). WAMI was considered appropriate because we were specifically interested in understanding the experiences and processes that give rise to meaningful work, which may be better assessed by multidimensional scales such as WAMI (Allan et al., 2019). In addition, various studies have referred to WAMI as an appropriate measurement model for understanding meaningful work experiences in the context of sensemaking (Aguinis & Glavas, 2019) and specifically in the context of the pandemic (Kramer & Kramer, 2020). The WAMI measures meaningful work through the three dimensions discussed earlier; positive meaning (PM; 4 items), broader meaning making (MM; 3 items), and greater good motivation (GG; 3 items). Sample items include: “I have found a meaningful career” (PM), “My work helps me better understand myself” (MM), and “The work I do serves a greater purpose” (GG), measured from 1=strongly disagree to 7=strongly agree. Again, at the beginning of this part of the survey, respondents were prompted to consider their work experiences during the pandemic.

*Mental health* was assessed through the Mood and Anxiety Symptom Questionnaire-D30 (MASQ-D30: Wardenaar et al., 2010), a 30-item set of questions measuring three subscales, including general distress (e.g., “I felt hopeless”), symptoms of depression (lack of positive affect) (e.g., “I felt like I was having a lot of fun” (reversed)), and anxiety (e.g., “I was trembling or shaking”). To ease interpretation, depression items were recoded such that higher scores indicate greater depression symptoms. Employees were asked to rate how much in the past week they have experienced these “feelings, sensations, problems and experiences that people sometimes have,’’ measured from 1=not at all to 5=extremely.

### Measurement Validation

As this study aims to study the influence of the COVID-19 prompted recategorization of workers into non-essential and essential, a multigroup analysis was performed. The factor loadings were invariant across groups (χ2_(48)_ = 58.85, *p* = .136), establishing pattern factorial invariance. However, strong factorial invariance was not established (χ2 (76) = 123.88, *p* < .001). However, this is neither important nor surprising as we do not expect the intercepts to be invariant across groups; the group differences in specific factors are indicative of individual differences relevant to the study. Mean comparisons indicate essential workers report significantly higher meaningful work (*M* = 5.23, *SD* = 1.06) compared to non-essential workers (*M* = 4.63, *SD* = 1.17, *t* = −6.693, *p* < .001). Similarly, essential workers report higher means on perceived disruptions (*M*_essential_ = 3.48, *SD* = 1.27, *M*_non-essential_ = 3.24, *SD* = 1.23, t = −2.321, *p* = .021) and anxiety (*M*_essential_ = 1.53 *SD* = 0.64; *M*_non-essential_ = 1.35, *SD* = 0.46, *t* = −3.979, *p* < .001), compared to non-essential workers.

As the factor loadings were established to be invariant across groups, we present the analysis of the measurement model using a single group CFA (see Table 1), as that improves the variable-to-observation ratio. The final model demonstrated adequate model fit: χ2_(1203)_ = 2923.65, CFI = 0.91, TLI = .90, SRMR = .05, PClose = .936, and RMSEA = .048 [CI: .046, .050]. The measurement model tested whether the perception of work as meaningful is best represented as a second-order factor. In line with the theory, anxiety, general distress, and anhedonic depression were used as three separate factors. This model fit better than a second-order construct for mental health Δ χ2_(3)_ = 33.07, *p* < .001.

**Table 1.** Model Validity Statistics.

Variable	M (SD)	CR	AVE	MSV	MaxR(H)	Range λ	1	2	3	4	5	6	7	8
1 Type of work^a^	0.48 (0.50)	—	—	—	—	—	—							
2 Disruption of work	3.36 (1.25)	.80	.36	.07	.83	.50–.82	.09	.60						
3 Change communication quality	5.16 (1.17)	.94	.72	.10	.95	.78–.92	.03	.12	.85					
4 Identity threat	2.90 (1.21)	.84	.51	.10	.86	.57–.81	.06	.13	−.29	.72				
5 Work as meaningful	4.92 (1.16)	.95	.87	.08	1.00	.45–.91	.26	.21	.27	−.10	.93			
6 Anxiety	1.44 (0.56)	.89	.45	.54	.90	.50–.74	.16	.13	−.10	.25	−.10	.67		
7 General distress	1.75 (0.71)	.89	.44	.54	.89	.49–.76	.06	.19	−.13	.29	−.17	.72	.66	
8 Anhedonic depression	3.40 (0.83)	.91	.50	.17	.92	.57–.83	−.05	.00	−.16	.12	−.26	.23	.35	.70

^a^*Note:*0 = non-essential work, 1 = essential work; CR = Composite Reliability; AVE = Average Variance Extracted; MSV = Maximum Shared Variance; MaxR(H)= Maximum Reliability. Square Root of the AVE appears on the diagonal. Correlations of .10 and higher are significant at *p* < .05.

For three concepts, the average variance extracted (AVE) was below .50: that is, for two dimensions of the MASQ-D30, anxiety (.45), general distress (.44), and the measure of perceived disruptions (.36). For the anxiety subscale, “I startled easily” (.50) and “I felt nauseous” (.58) contributed to a lower average variance extracted. However, these items were retained as they have very similar loadings as in the validation study by Wardenaar et al. (2010). For the general distress measure, two factor loadings were low: “I felt confused” (.56) and “I felt irritable” (.48). Again, these items were retained for their psychometric value; in addition, these loadings are similar to the those reported in the original validation study. The highest maximum shared variance (MSV) was between general distress and anxiety (.54); MSV between other concepts in the model ranged between .08 and .17. The inter-correlations between the concepts in our model ranged between −.29 and .72. Composite reliabilities (CR) ranged from .84 to .95, while the maximum reliability (H) ranged between .86 and 1, indicating good reliability overall.

## Results

**Figure 2.**
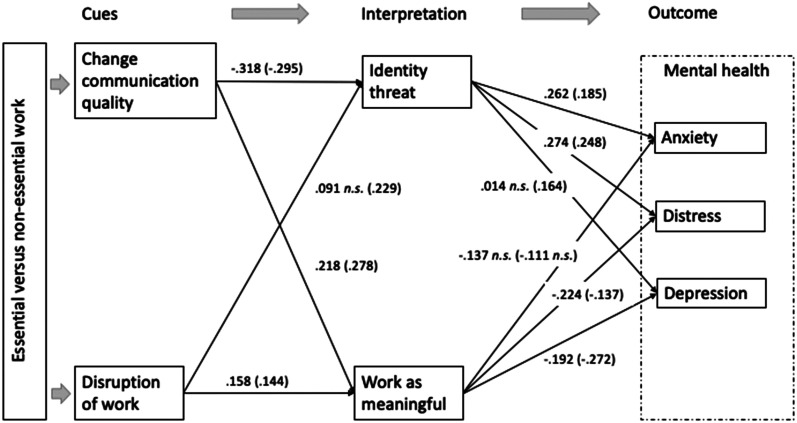
Structural model with standardized solutions. *Note:* Values without parentheses are standardized coefficients for employees conducting essential work; those in parentheses are for non-essential work employees. All are significant at *p* < .01 unless indicated otherwise.

The hypothesized structural model was tested using multigroup path modelling in AMOS (v 23). The results indicated good model fit: χ2_(12)_ = 33.97, CFI = .97, TLI = .90, SRMR = .05, Pclose = .338, and RMSEA = .054 [CI: .033, .076]. Table 2 displays the regression weights and confidence intervals for essential and non-essential workers. Figure 2 presents the standardized solution for the model parameters for all employees.

**Table 2.** Model Parameters for Essential and Non-Essential Workers.

	Essential workers						Non-essential workers					
	Bootstrapping BC 95% CI						Bootstrapping BC 95% CI					
	β	SE β	B	Lower	Upper	*p*	β	SE β	B	Lower	Upper	p
*H1a1* Disruption → Identity threat	.091	.058	.091	−.028	.219	.139	.229	.058	.214	.102	.315	.001
*H1b1* Disruption → Work as meaningful	.189	.070	.158	.039	.269	.008	.144	.049	.136	.041	.233	.005
*H2a* Change communication quality → Identity threat	−.318	.050	−.335	−.454	−.216	.001	−.295	.050	−.299	−.410	−.197	.001
*H2b* Change communication → Work as meaningful	.213	.059	.189	.090	.284	.002	.278	.059	.286	.159	.398	.001
*H3a* Identity threat → Anxiety	.262	.053	.132	.077	.191	.001	.185	.056	.073	.028	.124	.002
*H3b* Identity threat → General distress	.274	.051	.163	.100	.232	.001	.248	.051	.144	.083	.206	.001
*H3c* Identity threat → Anhedonic depression	.014	.055	.009	−.072	.091	.829	.164	.055	.121	.039	.196	.002
*H4a* Work as meaningful → Anxiety	−.137	.065	−.082	−.165	.002	.054	−.111	.065	−.043	−.097	.005	.069
*H4b* Work as meaningful → General distress	−.192	.057	−.136	−.233	−.039	.004	−.137	.057	−.078	−.144	−.018	.011
*H4c* Work as meaningful → Anhedonic depression	−.224	.052	−.170	−.271	−.053	.007	−.272	.052	−.197	−.279	−.127	.001

*Note:* BC = Bias Corrected; CI = Confidence Interval; SE β = standard error for standardized regression weight; 5,000 bootstrap samples.

We estimated several additional models to assess whether such alternative explanations generated equivalent or better fit to the data. First, re-specifying the hypothesized model as a CFA model—that is, estimating hypothesized relationships as non-directional unanalyzed associations between factors (i.e., covariances)—led to inferior model fit (*Δ*χ*2* = 17.73, *p* < .001). Second, a reverse causal model, based on the assumption that individuals’ mental health may affect information processing and thus, perceptions of communication quality and arguably disruptions, exhibited worse fit compared to the hypothesized model (*Δ*χ*2* = 54.54, *p* < 0.001). Third, we investigated three alternative mediation and moderation links. First, we estimated a simplified model treating disruptions, communication quality, work categorizations, and meaningful work as predictors, identity threat as mediator, and mental health as the outcome. This model showed significantly worse model fit compared to the hypothesized model (*Δ*χ*2* = 62.74, *p* < 0.001). Next, we estimated two models treating meaningful work as mediator in, or as moderator of, the relationship between communication quality and identity threat. Again, results demonstrated worse fit for the serial mediation model (*Δ*χ*2* = 18.65, *p* < 0.001), and for the moderation model (*Δ*χ*2* = 56.10, *p* < 0.001).

### Disruptions

**Figure 3.**
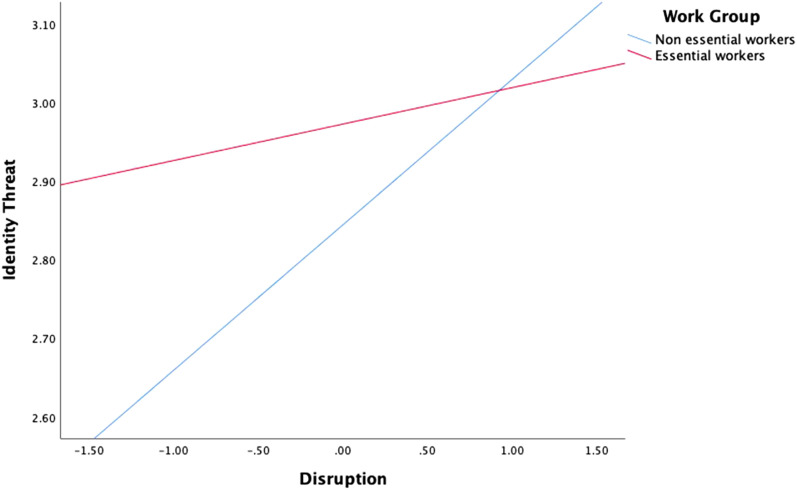
Interaction plot for the relationship between disruption and identity threat for non-essential and essential employees.

H1a1 addresses how workers in the two categories interpret the disruptions they experience in their work processes. Overall, disruptions were not significantly associated with identity threat (see Figure 2). However, while essential workers did not experience significantly higher identity threats due to disruptions in work (B_essential_ = .091, CI95% [−.028; .219], *p* = .139), non-essential workers did (B_non-essential_ = .214, CI95% [.102; .315], *p* = .001); and this relationship was significantly stronger for non-essential workers (*Ζ* = 1.677, *p* = .047). These results do not support H1a1 but do support the moderation of H1a2 (Figure 3).

Concerning H1b1, overall, disruptions of work were significantly associated with greater meaningfulness of work (see Table 2). More specifically, essential workers (B_essential_ = .158, CI95% [.039; .269], *p* = .008) experienced higher perceptions of meaningful work from such disruptions. However, contrary to our expectations, non-essential workers equally experienced disruptions as improving meaningful work perceptions (B_non-essential_ = .136, CI95% [.041; .233], *p* = .005) (*Ζ* = −0.319, *p* = .375), supporting H1b1 but not H1b2.

### Change Communication Quality

H2 proposes that the quality of change communication will reduce identity threat (H2a) and increase the perceptions of meaningful work (H2b). Figure 2 shows that both were supported overall. Change communication quality reduced identity threat for both essential and non-essential workers (B_essential_ = −.335, CI95% [−.454; −.216], *p* = .001; B_non-essential_ = −.299, CI95% [−.410; −.197], *p* = .001) (supporting H2a), and the difference was not significant across groups (*Ζ* = 0.452, *p* = .326) (not hypothesized). Further, there was a positive relationship between change communication quality and perceptions of meaningfulness of work for both groups (B_essential_ = .189, CI95% [.090; .284], *p* = .002; B_non-essential_ = .286, CI95% [.159; .398], *p* = .001) (supporting H2b), and the difference was not significant (*Ζ* = 1.321, *p* = .093) (also not hypothesized).

### Identity Threat

Overall, identity threat was positively related to anxiety and distress, but not significantly related to depression, providing partial support for H3 (see Table 2). Although we did not specify differential relationships between identity threat and mental health across the two work sectors, the relationships for identity threat on anxiety and on depression did differ across groups. For anxiety the impact of identity threat (H3a) was stronger for essential workers: (B_essential_ = .132, CI95% [.077; .191], *p* = .001; B_non-essential_ = .073, CI95% [.041; .124], *p* = .002; *Ζ* = −1.669, *p* = .048); while the relationship between identity threat and depression (H3c) was stronger for non-essential workers (B_essential_ = .009, CI95% [−.072; .091], *p* = .829; B_non-essential_ = .121, CI95% [.039; .196], *p* = .001; *Ζ* = 2.107, *p* = .018). There was no difference across groups in the relationship between identity threat and general distress (H3b): (B_essential_ = .163, CI95% [.100; .232], *p* = .001; B_non-essential_ = .144, CI95% [.083; .206], *p* = .001; *Ζ* = −.417, *p* = .093).

### Meaningfulness of Work

We examined the relationships between perceptions of work as meaningful and mental health (H4), indicated in Figure 2. Overall, meaningfulness of work was not significantly associated with anxiety (though the direction was negative). However, it was significantly associated with less distress and less depression. The relationship between work as meaningful and anxiety just failed to reach significance for either group (B_essential_ = −.082, CI95% [−.165; .002], *p* = .054; B_non-essential_ = −.043, CI95% [−.097; .005], *p* = .069; *Ζ* = 0.987, *p* = .162) (H4a). Perceptions of work as meaningful was, for both groups, negatively related to general distress (B_essential_ = −.136, CI95% [−.233; −.039], *p* = .004; B_non-essential_ = −.078, CI95% [−.144; −.018], *p* = .011; *Ζ* = 1.170, *p* = .121) (H4b), and to anhedonic depression (B_essential_ = −.197, CI95% [−.279; −.127], *p* = .001; B_non-essential_ = .121, CI95% [.039; .196], *p* = .001; *Ζ* = −0.465, *p* = .321) (H4c). Although not hypothesized, there were no significant differences in effect sizes across groups. Overall, these results provide partial support for H4.

### Indirect Effects on Mental Health

Finally, we examined the (conditional) indirect relationships as proposed in H5 and H6 in the form of moderated mediation relationships. Table 3 reports all indirect effects, for essential and non-essential workers, and for differences in indirect relationships between them. The indirect effects of disruptions, through identity threat, on all three aspects of mental health were insignificant for essential workers, while significant for non-essential workers (rejecting H5a1). The effect sizes of indirect effects were not significantly different between groups for anxiety and distress, but were significantly higher for depression through identity threat for non-essential workers (ΔB = −.025, CI95% [−.050; −.006], *p* = .013) (partially supporting H5a2). Hence, despite the significant interaction between work types and disruption on identity threat, the results provide only limited evidence for differences across work types on the moderated mediation relationships hypothesized H5a2. This is in part because the direct effect of disruption → identity threat was stronger for non-essential workers compared to essential workers, while the direct effect of identity threat → mental health issues was stronger for essential workers compared to non-essential workers, which mitigated the differences in the overall indirect effects. Concerning H5b about indirect effects of disruption through work meaningfulness on mental health, all of the three relationships (anxiety, distress, or depression) were significant (supporting H5b1), but none was significantly different between the two work categories (rejecting H5b2).

**Table 3.** Analysis of Indirect Effects.

	Essential workers				Non-essential workers				Contrast of indirect effects between essential and non-essential workers				
	Bootstrapping BC 95% CI				Bootstrapping BC 95% CI				Bootstrapping BC 95% CI				
Relationship	β	Low	Up	*p*	β	Low	Up	*p*		Δβ	Low	Up	*p*
*H5a1* Disruption → Identity threat → Anxiety	.012	−.003	.034	.104	.016	.005	.032	.001	*H5a2*	−.004	−.026	.018	.678
*H5a1* Disruption → Identity threat → Distress	.015	−.004	−042	.119	.031	.012	.056	.001	*H5a2*	−.016	−.049	.013	.256
*H5a1* Disruption → Identity threat → Depression	.001	−.006	.013	.635	.026	.026	.051	.001	*H5a2*	−.025	−.050	−.006	.013
*H5b1* Disruption → Work as meaningful → Anxiety	−.013	−.031	−.002	.022	−.006	−.006	.000	.045	*H5b2*	−.007	−.026	.007	.268
*H5b1* Disruption → Work as meaningful → Distress	−.021	−.047	−.047	.003	−.011	−.011	−.002	.007	*H5b2*	−.011	−.036	.008	.229
*H5b1* Disruption → Work as meaningful → Depression	−.027	−.058	−.058	.008	−.027	−.027	−.010	.003	*H5b2*	.000	−.034	.030	.974
*H6a* Change communication quality → Identity threat → Anxiety	−.044	−.075	−.022	.001	−.022	−.041	−.009	.001	—	—	—	—	—
*H6a* Change communication quality → Identity threat → Distress	−.054	−.090	−.030	.001	−.043	−.071	−.025	.000	—	—	—	—	—
*H6a* Change communication quality → Identity threat → Depression	−.003	−.032	.025	.827	−.036	−.069	−.012	.002	—	—	—	—	—
*H6b* Change communication quality → Work as meaningful → Anxiety	−.016	−.037	−.001	.036	−.012	−.033	−.000	.054	—	—	—	—	—
*H6b* Change communication quality → Work as meaningful → Distress	−.026	−.054	−.008	.003	−.022	−.048	−.005	.008	—	—	—	—	—
*H6b* Change communication quality → Work as meaningful → Depression	−.032	−.064	−.009	.005	−.056	−.098	−.029	.000	—	—	—	—	—

*Note:* Bolded values indicate non-significant relationships or differences. Contrast effects represent a test of moderated mediation by comparing the effect sizes of indirect effects for essential workers to those for non-essential workers.

Finally, H6a and H6b test the assumption that change communication quality is negatively related to mental health through decreased identity threat, and increased meaningfulness of work. The findings largely support these hypotheses, as the indirect effects were all significant for essential and non-essential workers for identity threat (H6a) and for meaningfulness of work (H6b), although not for the indirect effects of change communication quality on depression through identity threat for essential workers (B_essential_ = −.003, CI95% [−.032; .025], *p* = .827).

## Discussion

This study heeds recent calls to examine differential impacts of the crisis depending on occupational groups or status (Spurk & Straub, 2020), especially the question of how “people sensemake around labels of essential and nonessential work” (Stephens et al., 2020, p. 444). The results provide insights into the impact of the COVID-19 pandemic in terms of work disruptions and change communication—and associated categorizations of work types as non-essential or essential—on three aspects of employees’ mental health through identity threat and perceptions of meaningfulness of work. The results suggest that the disruptions and change communication provide cues for a sensemaking process that leads essential and non-essential workers to the situational re-evaluation of their professional identities and the meaningfulness of work. Disruptions of work due to COVID can help to reduce mental health problems by emphasizing work as a source of meaning, while it may increase mental health concerns when it triggers identity threat, especially for workers newly categorized as conducting non-essential work. Change communication quality is important, as it provides cues that may reduce identity threats and increase perceptions of work as meaningful, as such reducing mental health issues for both essential and non-essential workers.

### Theoretical Implications

The findings have important theoretical implications. First, they contribute to literature on sensemaking in organizations during times of crisis. Several studies and essays have devoted attention to sensemaking during the COVID-19 pandemic, highlighting its importance for policy decision making (Angeli & Montefusco, 2020), the role of leadership in sense-giving processes (Crayne & Medeiros, 2020), and collective sensemaking more broadly (Christianson & Barton, 2020; Stephens et al., 2020). This is not surprising because crises, especially of this magnitude, are prototypical of the ambiguous, high-impact events for which sensemaking is most needed (Crayne & Medeiros, 2020). Sensemaking has long been viewed as entangled with issues of identity and meaning, especially during crisis and change (Maitlis & Christianson, 2014). The findings represent a sensemaking process by demonstrating that essential and non-essential employees somewhat differentially interpret the meaning of environmental cues in terms of identity threats and meaningfulness, which ultimately affects mental health outcomes.

The results further highlight the importance of change communication, evidenced by its positive relationship to meaningful work perceptions and the negative implications on perceived identity threats. Interestingly, these implications hold true across both work categorizations. In line with Li et al. (2021), these findings demonstrate that change communication quality during crises can influence the ways employees interpret, manage, and cope with the situation. This may not only have a positive impact on employee-organization relationships (Ecklebe & Löffler, 2021) but also on the social identities of employees. It is particularly important that marginalized groups in organizations (and society)—for instance those who might be considered less essential to the organizations’ core processes—also have access to transparent organizational communication, involving relevant, needed, substantial, accurate, timely, and balanced information.

In addition, research on sensemaking suggests that reducing uncertainty, ambiguity, and complexity has beneficial outcomes. However, sensemaking is also an effortful process (Christianson, 2019), which may deplete resources (Christianson & Barton, 2020), potentially leading to reduced wellbeing. Yet, individual sensemaking may also be motivated by a need for social connection and reassurance. The interpretative frames of employees categorized as conducting non-essential work may lead them to interpret disruptions as a threat to their social identities; this relationship was not evident for employees with essential work. However, perceptions of disruptions do not affect work as meaningful differently for essential and non-essential employees. The positive relationships found in this study suggest that breaking routines, interrupted interactions, and information processes during COVID-19 may lead to reevaluating the meaningfulness of work regardless of the type of work.

This study also provides insights regarding how to conceptualize these disruptions. The results showing that disruptions of work positively affect meaningful work, for both the essential and the non-essential worker, imply that disruptions can be viewed as challenges providing opportunities to demonstrate competence, achievement, and gain meaningfulness (Kim & Beehr, 2020). Meaningfulness may be particularly triggered in situations (such as during the COVID-19 crisis) where employees are facing challenges that provide opportunity for learning, high achievement, and future gains. Indeed, Steger et al. (2012) argue that the positive valence of meaningful work has a eudaimonic rather than hedonic focus, suggesting that, much like challenge demands, it is growth- and purpose-oriented.

Finally, much research on mental health issues during global health crises has focused on essential frontline and particularly medical personnel (Du et al., 2020), or the general public more broadly (Saltzman et al., 2020). However, these studies do not consider how employees in non-essential work across industries and organizations have been affected compared to essential workers (who often conduct work on the health frontline). In addition, most of these studies have focused on how uncertainty, the physical threat associated with the virus (e.g., threat of infection), and work pressure have increased mental health issues of health care workers, and how loneliness, isolation, and a lack of social support may be detrimental to the public’s mental health (Saltzman et al., 2020). This study demonstrated that essential and non-essential workers’ mental health may be improved when change communication quality is high, and when disruptions triggered by the crisis are viewed as challenges that afford opportunities for achievement, rather than as hindrances. Nonetheless, this study showed that non-essential workers may not only need to cope with isolation or lack of social support but may also experience increased mental health issues—that is, anxiety, distress, and depression—due to increased threats to their professional identities.

### Practical Implications

Interestingly, business reports reflect the assumption that organizations will not return to business as usual (e.g., Caglar et al., 2020). Disruptions will continue to affect organizations and work processes as additional lockdowns, relaxations of measures, multiple waves of new outbreaks, and possible future pandemics, will continuously require organizational and individual agility. Our findings suggest that these disruptions may not always have a detrimental impact on individuals as they can also present an opportunity to reflect and recommit by finding meaning in work, in turn reducing mental health issues. However, these disruptions need to be carefully managed, as some employees may also interpret these disruptions, and work recategorizations, as identity threats. Therefore, organizations should consider how they can maintain an inclusive work climate among a remote and dispersed workforce that may not be equally affected by workplace disruptions.

The findings also highlight the importance of providing high quality change communication. Importantly, for essential and non-essential workers, change communication can serve as a guide to sensemaking, from which employees derive meaning about their work, which in turn results in lower levels of mental health issues. The pandemic has generated a great deal of uncertainty as organization and employees were forced to transform their operational routines almost overnight (Sanders et al., 2020). In such times of crisis, effective communication is crucial as employees turn to organizational leaders for guidance and information. Though it might be easier said than done to provide high quality change communication, our findings provide an excellent starting point. As such, organizations should communicate clearly and frequently and, when doing so, connect to a deeper sense of purpose and stability, as well as distil meaning from chaos (Mendy et al., 2020). Especially focusing on a clear vision for how the organization and its members will emerge from the crisis may provide useful guidance to employees (Holtom et al., 2020). In addition, these types of communication may strengthen a shared identity, helping to reinstate the workplace as a powerful source of both organizational and professional identity.

### Limitations and Future Research

Notably, the study comes with several limitations. The research design was set up to examine differences between employees in essential and non-essential sectors; as such we sought a sample of workers across a variety of organizations and industries categorized into these sectors. We also relied on measures of employee interpretations of organizational change communication, rather than relational and communication dynamics between workers. We do not have information on any specific organizational context or the content of any of the change communication; thus, we cannot claim that the intricacies outlined here extend to every organizational or cultural context. Future research may examine *in situ* responses and experiences from workers with different occupational statuses within the same organizational context (Spurk & Straub, 2020; Stephens et al., 2020).

This study relies on cross-sectional data and was collected in the early stages of the COVID-19 outbreak response measures implemented by the Dutch government. The cross-sectional nature prevents any casual inferences, while initial responses to lockdown measures may have been particularly severe. Of course, from a perspective of sensemaking under pressure, this context is highly relevant. However, a longitudinal design would have allowed analysis of how these processes evolved. In addition, such research designs would be better suited to explore dynamic aspects related to a sensemaking perspective, such as feedback loops and employee interactions. For instance, research could consider how interpretations of meaningful work or identity threat triggered by disruptions and change communication may influence subsequent perceptions of disruption and communication throughout the crisis. Also, the long-term impact of these sensemaking processes in relation to wellbeing remains unclear. Furthermore, alternative causal orders may be examined; for instance, it is possible that mental health enables or deters information processing and therefore perceptions of communication quality and perceived disruptions, although these explanations seem less likely based on the alternative models we presented.

Third, the global COVID-19 crisis is complex and highly impactful for all aspects of individuals’ lives, including work and home. We included organizational communication due to its relevance to work-related outcomes such as identities and meaningfulness of work. However, throughout the crises, there are many different sensemaking and sensegiving entities ranging from governments, global entities (e.g., WHO), news media, social media, and various experts. Future research may examine a broader range of communication sources to better address the situated and interrelated nature of sensemaking (Christianson & Barton, 2020). This study serves as a starting point by contributing to organizational literature on how sense is made in organizations (Rudolph et al., 2009) by expanding the current focus of sensemaking on topics such as strategic change (Rerup & Feldman, 2011), organizational learning (Christianson et al., 2009), and interpersonal dynamics (Wrzesniewski et al., 2003), to include communicative cues and mental health outcomes in the COVID-19 context through sensemaking about the professional identities and meaningfulness of work.

## Notes

1.
https://www.cbs.nl/nl-nl/faq/corona/economie/hoeveel-mensen-werken-er-in-cruciale-beroepen-

2.
https://www.government.nl/topics/coronavirus-covid-19/childcare-for-children-of-people-working-in-crucial-sectors
